# Bioinspired Thermosensitive Hydrogel as a Vitreous Substitute: Synthesis, Properties, and Progress of Animal Studies

**DOI:** 10.3390/ma13061337

**Published:** 2020-03-15

**Authors:** Amine Laradji, Ying-Bo Shui, Bedia Begum Karakocak, Lynn Evans, Paul Hamilton, Nathan Ravi

**Affiliations:** 1 Department of Ophthalmology and Visual Sciences, Washington University School of Medicine, St. Louis, MO 63110, USA; alaradji@wustl.edu (A.L.); shui@wustl.edu (Y.-B.S.); karakocakb@wustl.edu (B.B.K.); lynn.evans@wustl.edu (L.E.); hamiltonpd@wustl.edu (P.H.); 2 Department of Veterans Affairs, St. Louis Medical Center, St. Louis, MO 63106, USA; 3 Department of Energy, Environmental, and Chemical Engineering, Washington University in St. Louis, St. Louis, MO 63110, USA

**Keywords:** vitreous, hydrogel, biomimetic, biocompatible, thermoresponsive, sol-gel transition, vitrectomy

## Abstract

In many vitreal diseases, the surgeon removes the natural vitreous and replaces it with silicone oils, gases, or balanced salt solutions to fill the eyeball and hold the retina in position. However, these materials are often associated with complications and have properties that differ from natural vitreous. Herein, we report an extension of our previous work on the synthesis of a biomimetic hydrogel that is composed of thiolated gellan as an analogue of type II collagen and poly(methacrylamide-co-methacrylate-co-bis(methacryloyl)cystamine), a polyelectrolyte, as an analogue of hyaluronic acid. This thermosensitive hydrogel can be injected into the eye as a viscous solution at 45 °C. It then forms a physical gel in situ when it reaches body temperature, and later forms disulfide covalent crosslinks. In this article, we evaluated two different formulations of the biomimetic hydrogels for their physical, mechanical, and optical properties, and we determined their biocompatibility with several cell lines. Finally, we report on the progress of the four-month preclinical evaluation of our bio-inspired vitreous substitute in comparison to silicone oil or a balanced salt solution. We assessed the eyes with a slit-lamp examination, intraocular pressure measurements, electroretinography, and optical coherence tomography. Preliminary results are very encouraging for the continuing evaluation of our bio-inspired hydrogel in clinical trials.

## 1. Introduction

The vitreous is an acellular transparent hydrogel that occupies two-thirds of the total volume of the eye. Although it is a gelatinous structure, water, both bound and free, constitutes approximately 98–99% of the vitreous, with the remainder being mainly collagen type II fibrils that are interspersed with hyaluronic acid chains [1]. These two components are primarily responsible for the osmotic pressure that gives the eye its spherical shape and holds the retina in place [2]. Vitrectomy, which is the removal of some or all of the natural vitreous, is often used in the treatment of several ophthalmological pathologies such as retinal detachment and diabetic vitreous hemorrhage. During or after vitrectomy, a vitreous substitute is needed to reattach or hold the retina in place. Currently used vitreous substitutes include silicone oil [3,4], saline buffers [5], expansible gases [6], and perfluorocarbons [7]. Each of these substitutes, however, comes with limitations and is associated with complications, such as emulsification for silicone oils, expansion of gases at high altitudes, and toxicity of perfluorocarbons. In addition, all of these substitutes noticeably alter the refractive index in the eye [8,9,10]. Therefore, there is a need for novel vitreous substitutes that are biocompatible and have similar physical and optical properties to the natural vitreous.

Polymer-based hydrogels have emerged as promising biomaterials that hold great potential in several biomedical fields, due to their high water content, optical clarity, and rheological properties [11,12,13]. Nevertheless, their use as a vitreous substitute has failed because the hydrogels, which are made before injection, shear-degrade when pushed through the small-gauge needle, changing their mechanical properties [2,14,15].

Our approach is designed to match the physical and mechanical properties of the natural vitreous by developing a biocompatible biomimetic vitreous substitute. When injected at 45 °C as a viscous solution, our substitute almost instantly forms a physical hydrogel in-situ upon cooling to a body temperature of 37 °C.

Several research groups have investigated the use of in situ forming hydrogels as vitreous substitutes. Some of the researched materials are hyaluronic acid, polyethylene glycol, and polyvinyl alcohol [16,17,18,19,20,21]. The first two polymers are prone to rapid degradation through oxidative damage, and the last one needs further study to show its long term stability and biocompatibility [22]. For all three substitutes, however, gelation is not instantaneous, which may result in diffusion of the polymer solution out of the eye cavity before gelation. Stimuli-responsive materials, on the other hand, can instantaneously form a three-dimensional structure in response to external stimuli [23]. Examples of early work in this direction are smart hydrogels made from Pluronic F127 and WTG-127 [24,25]. Despite their desirable physical and mechanical properties, the use of Pluronic F127 resulted in severe retinal toxicity, while using WTG-127 was associated with low stability and diffusion under the retina before gelation was complete [22].

Inspired by the natural composition of the vitreous, we here report on the development of a two-component vitreous substitute that mimics the essential properties of the natural vitreous. The two-component vitreous substitute is made of gellan, an analogue of collagen, which undergoes a sol-gel transition at a defined temperature in the presence of cations to form a physical hydrogel. The mechanism of gelation consists of a random coil-to-double-helix transition when the temperature is decreased below the transition temperature of gellan [26]. The other component is poly(methacrylamide-co-methacrylate-co-bis(methylacryloyl-cystamine)), (poly(MAM-co-MAA-co-BMAC), an analogue of hyaluronic acid. This copolymer is an anionic polyelectrolyte whose role is to control the osmotic pressure and sol-gel transition temperature of gellan. The monomer BMAC was copolymerized to introduce thiol groups through the copolymer backbone. Under physiological conditions, thiol groups oxidize and undergo interchain chemical crosslinking. This step is essential to avoid diffusion of the hydrogel, as previously observed for other polymers [14]. The other benefit of crosslinking through disulfide bond formation is the possibility of reversing the oxidation by using a reducing agent, such as glutathione or DTT, and of removing the vitreous substitute without requiring a second surgery. In this study, we aim to report the progress of the evaluation of an in-situ-forming two-composite hydrogel in preclinical research and compare its biocompatibility against silicone oil (positive control) and a buffered salt solution (negative control). Silicone oils are the current standard of care for large retinal detachment.

## 2. Experimental Section

### 2.1. Materials Synthesis and Characterization

We determined the purity of all monomers and the initiator for the free radical polymerization reaction by quantitative nuclear magnetic resonance (qNMR). An NMR standard certified by the National Institute of Standards and Technology (NIST) was used. The moisture content of the gellan was determined by a gravimetric analysis comparing its weight before and after moisture removal.

^1^H NMR spectra were recorded for poly(MAM-co-MAA-co-BMAC) and thiolated gellan by Varian Unity Inova spectrometer (400 MHz, Agilent Technologies, Santa Clara, CA, USA), using D_2_O as a solvent. A Bruker Avance III^TM^ HD 600 MHz NMR spectrometer (Billerica, MA, USA) was used to determine the materials’ purity.

The molecular weight of poly(MAM-co-MAA-co-BMAC) was measured by gel permeation chromatography (Viscotek GPC system from Malvern PANalytical, Westborough, MA, USA) using a TDA302 triple detector system (refractive index, multiangle laser light scattering, and viscosity). Samples were injected at a concentration of 2 mg/mL, and the flow rate was 0.8 mL/min. All measurements were conducted at 37 °C.

The thiol content in thiolated gellan and poly(MAM-co-MAA-co-BMAC) was measured by 2-nitro-5-thiosulfobenzonate (NTSB) assay, as previously reported [27]. An Abbe refractometer (ATAGO Abbe Refractometer NAR-1T, Bellevue, WA, USA) was used to determine the refractive index of the hydrogels at 37 °C and 552 nm.

The transmittance of light between 200 and 800 nm was measured by a Thermo Scientific BioMate 3 UV/Vis spectrophotometer (Waltham, MA, USA). The evaluation of the physical and mechanical properties of the hydrogels was done as reported earlier [28,29]. We performed all measurements on hydrogels that were allowed to covalently crosslink for ten days. Briefly, for 3 mL of hydrogels, we added 1.5 mL of phosphate buffer saline (PBS) buffer 1X and allowed them to swell to equilibrium for one week. The swollen hydrogels were patted on a tissue paper and dried to remove excess fluid. We used a gentle vortex to remove any trapped air bubbles. We performed all measurements in triplicate, at room temperature (23 °C) for transmittance and density, and 37 °C for the refractive index.

For the synthesis of poly(MAM-co-MAA-co-BMAC) and thiolated gellan, a detailed description is provided elsewhere [28,29]. Two hydrogel formulations were prepared; the first one was made by mixing 1.5 mg/mL thiolated gellan with 10 mg/mL poly(MAM-co-MAA-co-BMAC), here noted as 1.5G_10Cop. In the second formulation, we mixed 0.9 mg/mL thiolated gellan with 12 mg/mL poly(MAM-co-MAA-co-BMAC), here noted as 0.9G_12Cop. 

### 2.2. In Vitro Biocompatibility Testing

In vitro biocompatibility testing was achieved by using electrical cell-substrate impedance sensing ECIS (Applied BioPhysics, Troy, NY, USA) as previously reported [29]. Thiolated gellan was dissolved in sterile N_2_-bubbled water, the poly(MAM-co-MAA-co-BMAC) was dissolved in sterile 2X Dulbecco’s Modified Eagles’ Medium/Nutrient Mixture F-12 Ham (DMEM/F12) media, and the pH was adjusted to 7.4 with 1M NaOH. The thiolated gellan and poly(MAM-co-MAA-co-BMAC) solutions were then heated separately at 45 °C for 15 min in a water bath and mixed immediately before exposure to the cells. The hydrogel solution was cast over the cells to ensure direct contact. In vitro biocompatibility testing was monitored using electrical cell-substrate impedance sensing (ECIS from Applied BioPhysics, Troy, NY, USA). The ECIS is a non-invasive technique that measures the impedance across gold electrodes at the bottom of tissue culture wells, using a range of frequencies of alternating current. After the initial seeding, the cells start to grow over the gold electrodes and increase the resistance to current flow; therefore, the impedance across the electrodes increases. In response to stimuli (in this case, the hydrogels), the degree to which cells are attached to the electrodes may change. This change is reflected in the impedance recordings in real-time. The improved biocompatibility is interpreted as an increase in impedance compared to the control cells, which are not exposed to any vitreous substitutes. 

Two sets of experiments were performed in this work. The first aimed to determine the biocompatibility of the hydrogels on a confluent layer of cells. Confluence was obtained by plating the primary porcine retinal epithelial cells (ppRPE) at a high cell density of 40,000 cells/well; human retinal pigment epithelial (ARPE-19) cells at 20,000 cells/well, and 3T3/NIH (fibroblasts) cells at 10,000 cells/well in an ECIS cultureware, 96-well plate. Upon confirmation of confluency, the polymer solution was added. In the second set of experiments, the cells were plated at low density (ppRPE at 10,000 cells/well, ARPE-19 at 5000 cells/well, and 3T3/NIH at 4000 cells/well), and the polymer solution was added the following day to investigate the proliferation of cells in the presence of the gel.

The CellTiter–Glo assay (Promega, Madison, WA, USA), which determines the number of viable cells based on ATP quantitation, was done according to the manufacturer’s instructions. The toxicity of the hydrogel formulations was tested on 3T3/NIH and primary porcine retinal epithelial (ppRPE) cells at two different cell seeding concentrations: proliferating (low cell density) and confluent (high cell density). For the biocompatibility experiments, the low and cell density numbers were used as determined in our previous studies [29,30,31]. The ppRPE cells were plated at a cell density of 40,000 cells/well, and the 3T3/NIH (fibroblasts) cells at 10,000 cells/well for the high cell density experiments. On the other hand, for the low cell density experiments, the cells were plated at 4000 cells/well and 10,000 cells/well for 3T3/NIH cells and ppRPE cells, respectively. 

The ARPE-19 and 3T3/NIH cell lines were purchased from American Type Culture Collection (Manassas, VA, USA). The ppRPE cells were cultured and seeded, as previously described [29,31].

### 2.3. Animal Studies

All animal studies were approved and guided by the Washington University School of Medicine (WU) and the United States Department of Defense (US DoD, protocol number 20180105, approval date 05/23/2018). Over several months, we completed 11 two-port partial pars plana vitrectomy surgeries on Dutch belted rabbits. Among these, five of the rabbits have reached the four-month observation time point. One rabbit received the silicone-oil vitreous replacement, two rabbits received a buffered salt solution (BSS) replacement, and two received the hydrogel substitute form the 1.5G_10CoP formulation.

The ocular status of the rabbit eyes were examined by electroretinography (ERG, RETevet, Gaithersburg, MD, USA), optical coherence tomography (OCT, Standard-Resolution 800 nm OCT System, Wasatch Photonics, Morrisville, NC, USA), slit-lamp examinations (PSL Classic Portable Slit Lamp, Keeler, Malvern, PA, USA) and intraocular eye pressure (IOP) measurements (Tono-Pen AVIA Tonometer, Reichert Technologies, Depew, NY, USA). These evaluations were performed before the start of surgery, at the one-month mark, and the four-month endpoint.

The surgeries were performed under general anesthesia, and vitals were monitored by the Department of Comparative Medicine (DCM) veterinarian staff. The surgical procedure was as follows: the rabbits were prepped and draped in a sterile surgical environment. The right eye (surgical eye) of each rabbit received a randomly chosen substitute to be placed in the eye.

**Figure 1:**
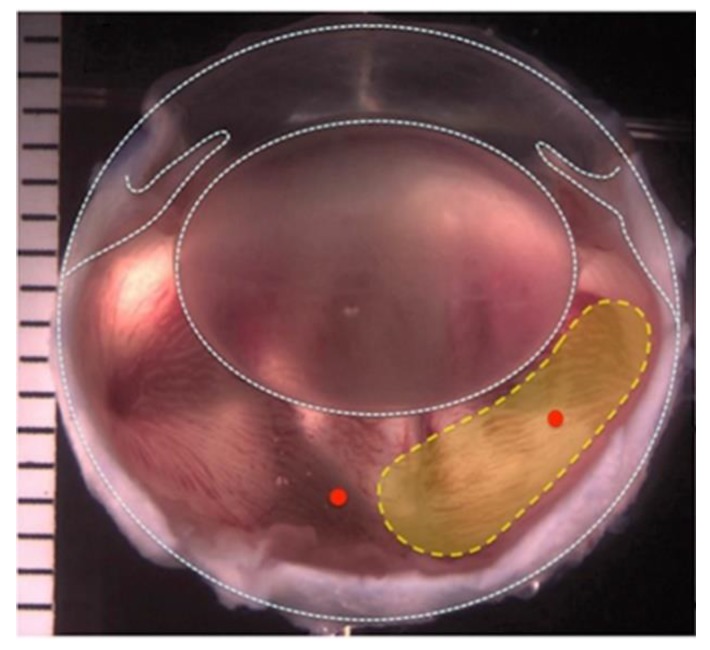
Image of a rabbit’s eye. Yellow region indicates the area where the natural vitreous was removed during the two-port partial pars plana vitrectomy.

Two 23-gauge trocars were inserted into the vitreous cavity from the temporal side, 2 mm from the limbus; one trocar was used for infusion of balanced salt solution (BSS), and the other to access the vitreous cavity. A 23-gauge vitrector probe (Millennium, Bausch & Lomb, San Dimas, CA, USA) was inserted into the rabbit’s temporal vitreous chamber, and 0.3–0.4 mL of the vitreous gel was partially removed, a fluid−air exchange was performed, and 0.3–0.4 mL of 1.5G_10Cop, silicone oil or balanced salt solution (BSS) was injected to replace the natural vitreous (Figure 1).

The left eye remained untreated as the control. Identical procedures were performed on rabbits who received silicone-oil and BSS. The rabbits received sub-conjunctival injections of gentamicin and dexamethasone to prevent post-operative infection and inflammation at the end of the surgical procedure. The IOP was measured immediately following the surgery. The IOP post-surgery ranged from 14–25 mmHg but was within normal limits the next day.

The surgical eyes were checked once or twice daily for up to one week. Depending on the eye’s condition upon slit-lamp examination, post-surgical treatments consisted of neomycin, polymyxin B sulfates, dexamethasone ophthalmic ointment (anti-fungal), gentamicin 0.3% ophthalmic solution (antibiotic), and atropine 1% ophthalmic solution (pupil dilation). The average recovery time following the surgery was one week.

ERGs were performed to measure the electrical activity generated by cells in the retina and to monitor the functioning of the retina with a three-step scotopic and two-step photopic protocol with flicker and flash testing. Additional information on the ERG analysis can be found in the Supplementary. The rabbits were placed in their cages under general anesthesia. Both eyes were dilated and dark-adapted for 20 min, then prepped with a viscous ophthalmic solution called Goniosol prior to insertion of an electrode on the cornea.

## 3. Results and Discussion

### 3.1. Synthesis and Properties of the Thermoresponsive Hydrogels

Thiolated gellan and poly(MAM-co-MAA-co-BMAC) (78% methacrylamide:20% methacrylic acid:2% bis-methacryloylcystamine) were synthesized according to our previously reported methods [28,29]. Using the NTSB essay [27] and ^1^H NMR analysis, we determined that thiolated gellan and poly(MAM-co-MAA-co-BMAC) have thiol contents of 11% (mol) and 2% (mol), respectively. The thiol content can be fine-tuned; however, we previously determined that increasing the thiol content negatively affects the biocompatibility of the hydrogel [28]. To determine the incorporation ratio of methacrylic acid, a solution of poly(MAM-co-MAA-co-BMAC) was titrated with a standard solution of 1 N NaOH. The incorporation ratio of methacrylic acid was found to be 19.31% ± 0.54, which is very close to the feed ratio of the monomer (20%). We measured the number average molecular weight of poly(MAM-co-MAA-co-BMAC) by gel permeation chromatography (GPC). Five batches of poly(MAM-co-MAA-co-BMAC) were synthesized, from which three were combined due to their similar molecular weights (311,256 g/mol ± 28,223).

In this study, two hydrogel formulations were synthesized by combining thiolated gellan and poly(MAM-co-MAA-co-BMAC) in different concentrations. In both formulations, the components were separately heated, then mixed at 55 °C. The sol-gel transition temperature was determined by a temperature scan from 55 to 15 °C, at a controlled cooling rate of 2 °C/min, following the loss modulus at 5% constant shear strain and 1.0 Hz constant frequency, using an Anton Paar rheometer MCR 302. As noted above, a good vitreous substitute should form a hydrogel in situ to avoid shear degradation when it is injected through a small gauge needle. However, failure to form a gel instantaneously may result in the polymers diffusing away from the injection site through retinal breaks [8]. Our hydrogels, however, are thermoresponsive; they can be injected at 45 °C as a viscous solution and form hydrogels almost immediately after they cool to body temperature. The sol-gel transition temperature of thiolated gellan at the studied concentrations falls in the range of 34–36 °C. The sol-gel transition temperatures of 1.5G_10Cop and 0.9G_12Cop are observed to be ~42 °C (Figure S12).

In addition to its effects in tuning the sol-gel transition temperature of thiolated gellan, poly(MAM-co-MAA-co-BMAC) also causes the hydrogels to swell and exert pressure, which is essential in holding the retina in place. The mixed thiolated gellan and poly(MAM-co-MAA-co-BMAC) were cast into 35-mL pre-weighed dishes, and the solutions were left to chemically crosslink via thiol oxidation at 37 °C for ten days in a humidified chamber. After chemical crosslinking, PBS (in the absence of Ca^2+^ and Mg^2+^) was added on top of the hydrogels. The swelling equilibrium was reached when no change in hydrogel’s weight was observed. The swelling percentages for each formulation were determined to be 17.74 ± 1.08% for 1.5G_10Cop, and 26.62 ± 2.06 for 0.9G_12Cop. A higher concentration of poly(MAM-co-MAA-co-BMAC) results in more swelling due to its ionic character and the chains’ semi-flexibility. Upon gelation, thiolated gellan forms a rigid double helix compact structure. This structure is able to control the swelling of poly(MAM-co-MAA-co-BMAC), forming a balanced vitreous similar to the natural vitreous, where the swelling of the ionic semi-flexible hyaluronan is confined by the rigid fibrillary collagen network [31].

The two swollen hydrogel formulations were investigated for their mechanical properties, refractive indexes, optical transmittances, and densities. Both hydrogel formulations exhibited physical and mechanical properties closely similar to the natural vitreous. The properties of natural tissues change with time, and the vitreous is no exception. For example, the viscoelastic properties of a pediatric eye had a higher elastic modulus compared to aged vitreous. Adult vitreous has an elastic modulus of 0.05–2.5 Pa [32,33]. The porcine vitreous is the closest to that of humans in volume and viscoelastic properties. It has a storage modulus of 0.3–8 Pa [34]. Similar to the natural vitreous, the storage modulus was higher than the loss modulus, indicating its solid-like viscoelastic behavior. In all viscoelastic solids, the storage modulus is higher than the viscous modulus. We tested our hydrogels at a constant strain of 2% at 37 °C, with a frequency scan that ranged from 10 to 0.01 Hz. The hydrogels had a storage modulus that ranged from 100–200 Pa as formulated.

The refractive index of natural vitreous is 1.3345–1.3348 [2]. The refractive index of our synthesized hydrogels ranged from 1.3355 ± 0.0003 for 0.9G_12Cop to 1.3370 ± 0.0001 for 1.5G_10Cop. While the refractive index of both our hydrogels is similar to the natural vitreous; however, it is much lower than the refractive index of silicone oil (1.4) [22], which is the currently used vitreous substitute. The density of the human vitreous lies within the range of 1.0053–1.0089 [2]. These values are very close to the density of our hydrogels, which is 1.003 for 0.9G_12Cop and 1.007 for 1.5G_10Cop.

**Figure 2:**
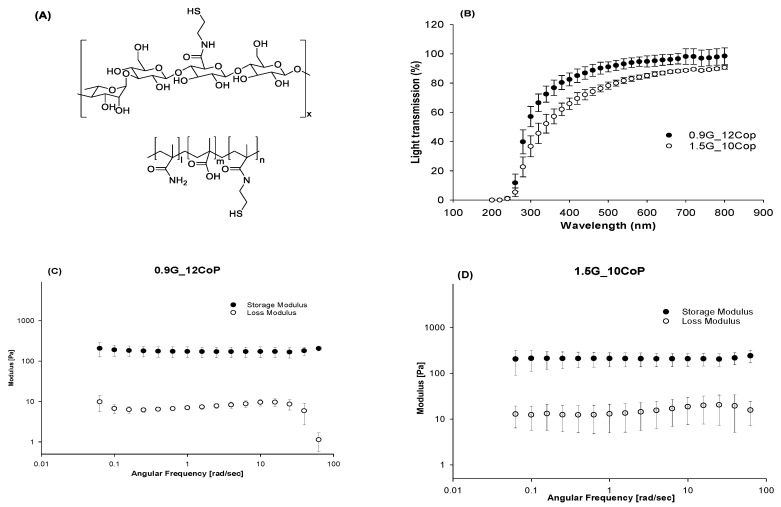
(**A**) Chemical structure of thiolated gellan and poly(MAM-co-MAA-co-BMAC); (**B**) Optical transmittance of hydrogel formulations between 200 to 800 nm; (**C**) Storage and loss moduli of 0.9G_12Cop formulation; (**D**) Storage and loss moduli of 1.5G_10Cop formulation.

For both formulations, we achieved an optical transmittance of more than 83% in the visible light range (Figure 2).

### 3.2. In Vitro Biocompatibility of the Formulated Hydrogels

**Figure 3:**
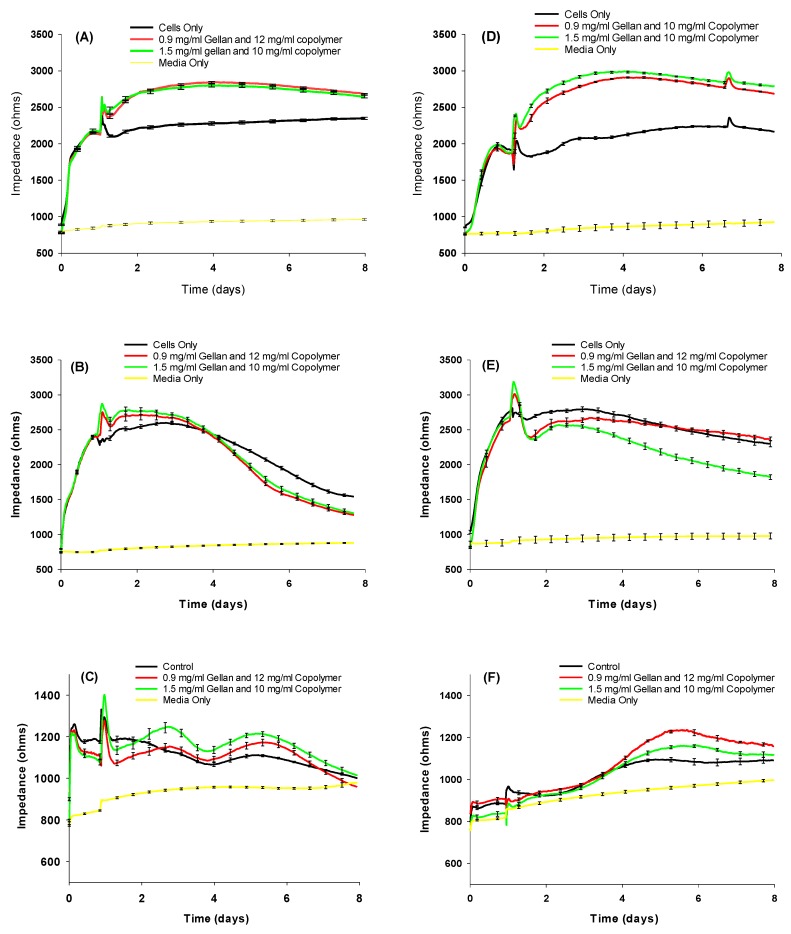
Resistance measurements with the two hydrogel formulations of confluent (**A**) APRE-19, (**B**) ppRPE, (**C**) 3T3 cells; and proliferating (**D**) ARPE-19, (**E**) ppRPE, and (**F**) 3T3/NIH cells. Values are given in mean ± SEM (n = 4).

To investigate their toxicity, the hydrogel formulations were tested on confluent and proliferating ARPE-19 cells, primary porcine RPE (ppRPE) cells, and 3T3/NIH cells (Figure 3). For biocompatibility testing, we used electric cell-substrate impedance sensing (ECIS), which allows for continuous testing over time using gold electrodes and a micro-electrical current. By measuring the impedance (Ohms) at an optimal frequency of 4000 Hz, we observed that the treatment with either formulation does not negatively affect tight junctions within cell monolayers. 

Compared to untreated cells, we observed that cell growth might be stimulated slightly upon the first addition of both materials. 

**Figure 4:**
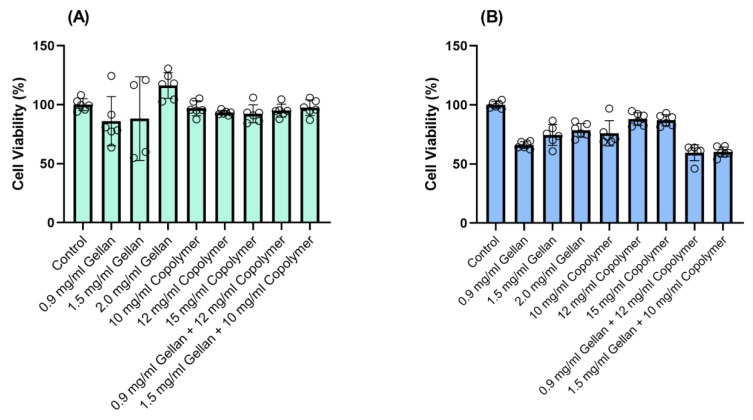
The viability of confluent (**A**) ppRPE, and (**B**) 3T3/NIH cells after exposure to thiolated gellan, poly(MAM-co-MAA-co-BMAC), and the combined components for 72 h. Values are given in mean ± SEM (n = 6).

The biocompatibility was further tested using a CellTiter-Glo Luminescent Cell Viability end-point assay (Figure 4), which is a standardized method for determining the number of viable cells in a culture based on quantitation of the ATP present, an indicator of metabolically active cells. For the biocompatibility analysis, we evaluated the statistical significance using analysis of variance (ANOVA) to compare the results with the respective negative (untreated cells) and group. P* < 0.05, the significance level, was statistically acceptable. 

For the biocompatibility experiments, ppRPE and 3T3/NIH (fibroblasts) cells were exposed to three concentrations of gellan (0.9-1.5-2.0 mg/mL) and copolymers (10-12-15 mg/mL), respectively. Finally, we tested the biocompatibility of the two formulations of our hydrogels on both cell types for 72 h. The results indicated that the two formulations were compatible with the ppRPE cells (Figure 4A). On the other hand, these two formulations slightly decreased the viability of the 3T3/NIH (fibroblasts) cells (Figure 4B).

Overall, our results indicate that both vitreous substitute formulations made from thiolated gellan and poly(MAM-co-MAA-co-BMAC), in addition to their individual components, are biocompatible with confluent and proliferating ARPE-19, pRPE, and 3T3/NIH cells.

### 3.3. Animal Studies

Pre-surgical ERGs showed overlapping latencies (time-to-peak) and amplitudes for the right eye and the left eye, indicating similar results for both eyes at the time of testing. Photopic b-wave and flicker amplitudes were similar for both eyes. The differences in summed oscillatory potential amplitudes may reflect electrical “noise” in some recordings.

**Figure 5:**
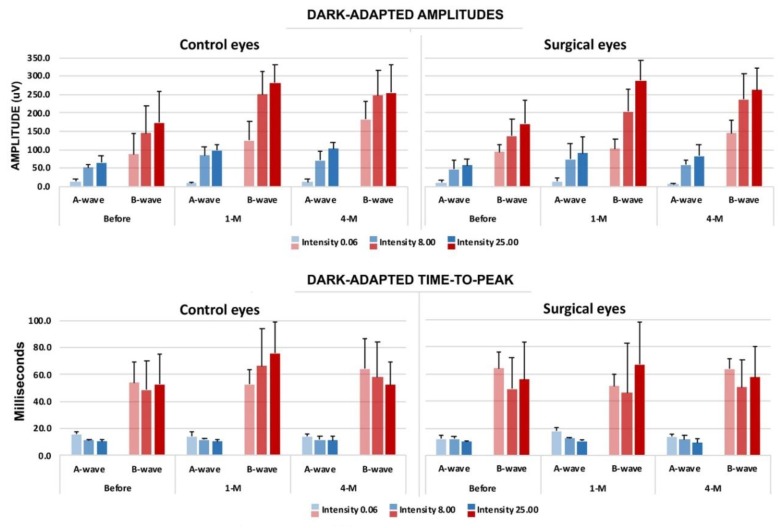
Evaluation results of electroretinography (ERG) with the time course compared to the surgical and non-surgical eye (negative control). Upper panel: dark-adapted amplitudes in both a- and b-waves. Lower panel: dark-adapted time-to-peak, both a- and b-waves. The blue bars indicate the a-wave, and the red bars indicate the b-wave. (n = 5.).

As seen in Figure 5, one-month post-operative ERGs show that, although there is some individual variation, especially in b-wave latency and amplitude, results from the control eyes show no significant difference from the surgical eyes. Amplitudes are substantially higher than in pre-surgical tests, indicating vision is likely being redefined or developed in the rabbits during the time between tests. 

Compared with the BSS and the silicone-oil groups at the four-month marker, the photopic and scotopic ERG analysis results did not show signs of toxicity from the rabbits tested in the 1.5G_10Cop formulation. The ERG analysis showed that the retina is functioning within normal limits in all three groups.

The OCT equipment was used to display high-resolution cross-sectional images of the retina. An ultra-compact probe was set 25 mm from the cornea, and the distance was adjusted according to the image presented on the monitor. Topical Goniosol was placed onto the cornea to prevent deterioration of the corneal epithelial layer.

**Figure 6:**
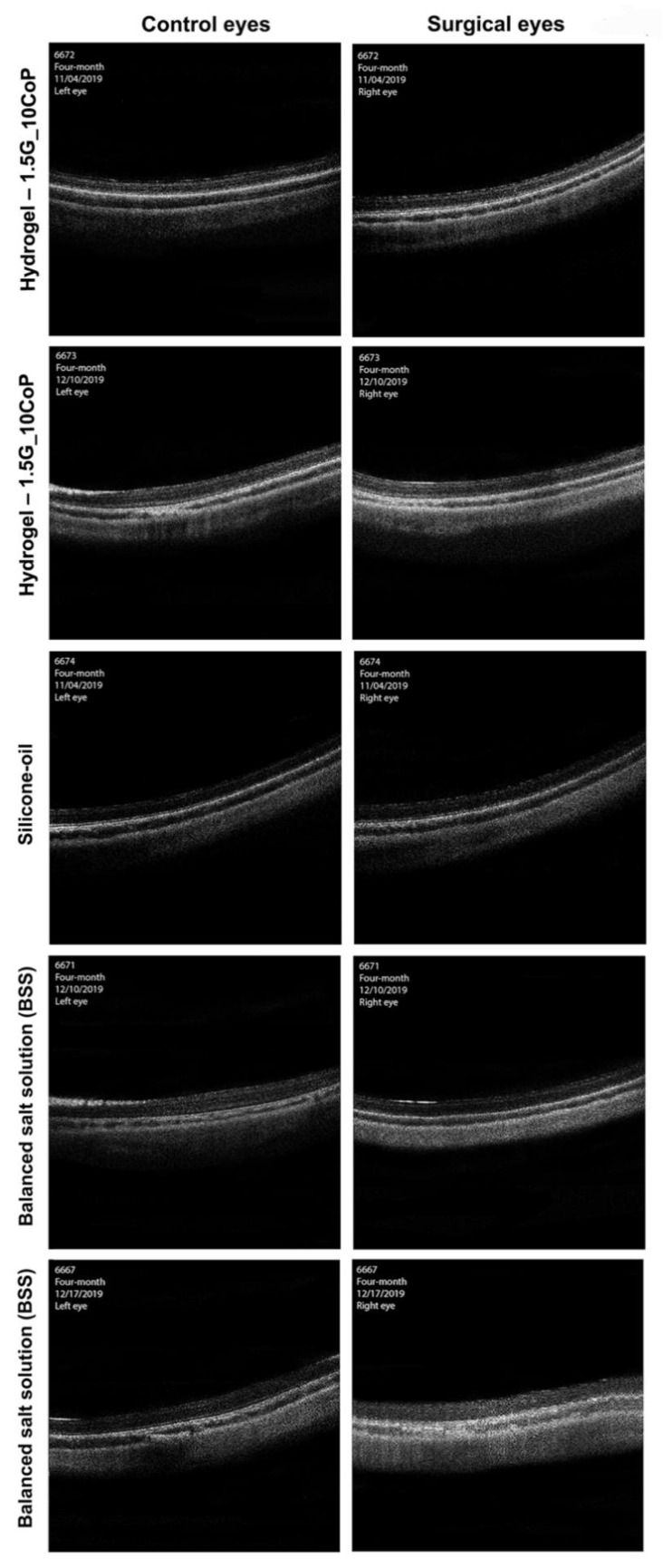
The optical coherence tomography (OCT) images were taken at four-month post-operative examinations. The OCT images on the left represent the control eyes, whereas the images on the right represent the surgical eyes.

The OCT imaging interpretation in Figure 6 shows no significant difference in the vitreous cavity or in the retinal layers of either the surgical or control eyes. At the four-month marker, the 1.5G_10CoP formulation shows the vitreous substitute is fully biocompatible in the Dutch belted rabbits and confirms the retina is functioning within normal limits. Additionally, there has been no evidence of retinal detachments in the OCTs. 

**Figure 7:**
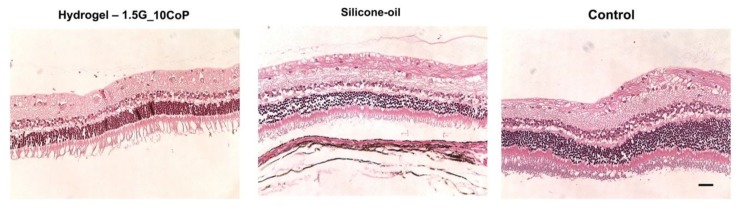
H&E staining on polyelectrolyte #1, silicone-oil, and control (BSS). The retina is detached from the pigmented layer. The retinal layers are clear, with no significant difference compared with the control (non-surgical eyes). The scale bar is 50 μm.

The five rabbits that reached the four-month marker were euthanized, and their eyes have been enucleated for histology analysis. Currently, we have obtained hematoxylin and eosin (H&E) staining slides of our hydrogel and silicone-oil eye substitutes along with the control eyes (Figure 7). The biggest concern is that during the histological preparations, all of the retinas have detached. Nevertheless, we know retinal detachments were not evident prior to enucleation, as observed and compiled from our OCT testing. 

From the hydrogel and the silicone-oil eye groups compared with the control, it is difficult to formulate a conclusion for the retinal morphology changes. However, we do not see significant differences thus far.

**Figure 8:**
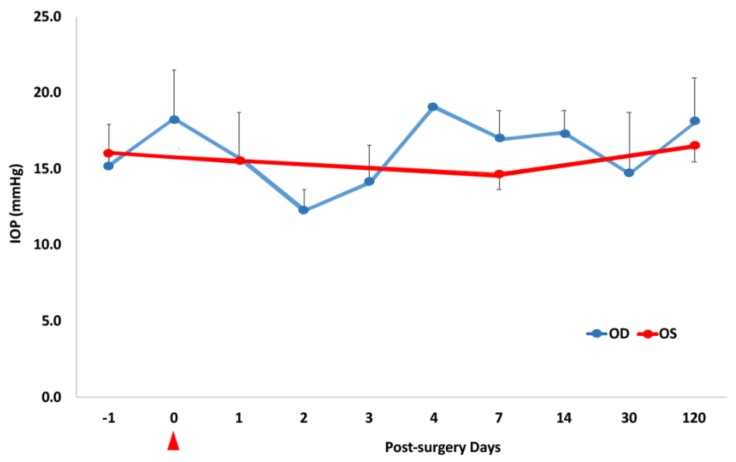
IOP changes graphed over time following the two-port partial pars plana vitrectomy. Red data points = control eyes; blue data points = surgical eyes. The red marker at 0 on the x-axis indicates the time immediately following the vitrectomy.

We used the Tono-pen AVIA Tonometer, a portable handheld device, to measure the rabbits’ intraocular eye pressure. Figure 8 shows the IOP measurement data for five rabbits, and all measurements are within normal limits. None of the rabbits developed high intraocular eye pressure or glaucoma as a result of the vitreous substitute surgical operation in vivo.

## 4. Conclusions

The present work reports the synthesis, properties, and progress of preclinical tests of two-component vitreous substitutes at two different concentrations. The vitreous substitutes were designed by reverse-engineering the features of the natural vitreous. In both formulations, the vitreous substitute exhibits thermoresponsive behavior that enables it to be injected as a viscous solution that then gels when it cools to body temperature. Following the initial, almost instantaneous gelling via physical crosslinking, the hydrogels start forming reversible chemical crosslinking through the oxidation of thiol to disulfide groups. Both formulations closely mimic natural vitreous. Toxicity studies on our vitreous substitutes reveal that they are biocompatible with several confluent and proliferating cell lines.

Preclinical studies on the four-month post-operative outcome of the two-port partial pars plana vitrectomy surgeries show no apparent signs or symptoms of inflammation in the anterior segment of the rabbits’ eyes. The rabbits’ corneas were transparent, and the anterior chambers were deep and quiet. There were partial opacities at the surgical site of the lens when viewed with the portable slit-lamp; otherwise, the lens was clear. Additionally, none of the rabbits in the four-month post-operative period developed a cataract. Further evaluation of the synthesized hydrogels as a vitreous substitute is ongoing, though current results suggest that it is a promising superior alternative to currently used materials.

## Supplementary Materials

The following are available online at https://www.mdpi.com/1996-1944/13/6/1337/s1. Figure S1. Reaction scheme of BMAC synthesis, Figure S2. ^1^H NMR spectra of the synthesized & purified BMAC, Figure S3. FTIR spectrum of the synthesized and purified BMAC, Figure S4. ^1^H NMR spectrum of AIBN and benzoic acid standard, Figure S5. ^1^H NMR spectrum of BMAC and benzoic acid standard, Figure S6. ^1^H NMR spectrum of MAM and benzoic acid standard, Figure S7. ^1^H NMR spectrum of MAA, before distillation, and benzoic acid standard, Figure S8. ^1^H NMR spectrum of MAA, after distillation, and benzoic acid standard, Figure S9. Reaction scheme of the copolymerization of MAM, MAA, and BMAC, Figure S10: ^1^H NMR of Poly(MAM-co-MAA-co-BMAC), Figure S11. Reaction scheme of gellan amidation, Figure S12: Temperature scan of thiolated gellan and thiolated gellan-copolymer, formulation 1.5G_10CoP, and 0.9G_12CoP, to determine the random coil-to-double-helix phase transition of thiolated Gellan (taken as the middle point of the loss modulus increase). The midpoint of the curves in both mixtures is ~42 °C. Finally, additional information on the ERG analysis is available.
