# Supplementary material for: Bioinspired Thermosensitive Hydrogel as a Vitreous Substitute: Synthesis, Properties, and Progress of Animal Studies
Source: Materials (Basel). 2020 Mar 15;13(6):1337. doi: 10.3390/ma13061337 (PMC7143394; doi:10.3390/ma13061337)
Supplement: Supplementary file 1 [file materials-13-01337-s001.pdf]

Supporting Information

Bioinspired Thermosensitive Hydrogel as a Vitreous Substitute: Synthesis, Properties, and Progress of Animal Studies

Amine Laradji [1,2], Ying-Bo Shui [1], Bedia Begum Karakocak [1,2], Lynn Evans [1], Paul Hamilton [1], and Nathan Ravi [1,2,3,*]

[1] Department of Ophthalmology and Visual Sciences, Washington University School of Medicine, St. Louis, Missouri 63110, United States
[2] Department of Veterans Affairs, St. Louis Medical Center, St. Louis, Missouri 63106, United States
[3] Department of Energy, Environmental, and Chemical Engineering, Washington University in St. Louis, St. Louis, Missouri 63110, United States

* Correspondence: ravi@wustl.edu

Synthesis of BMAC

BMAC synthesis was done according to the following scheme:

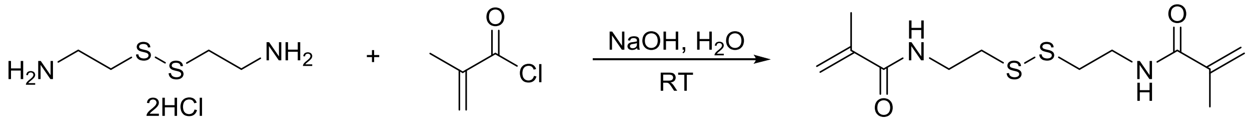

Figure S1. Reaction scheme of BMAC synthesis.

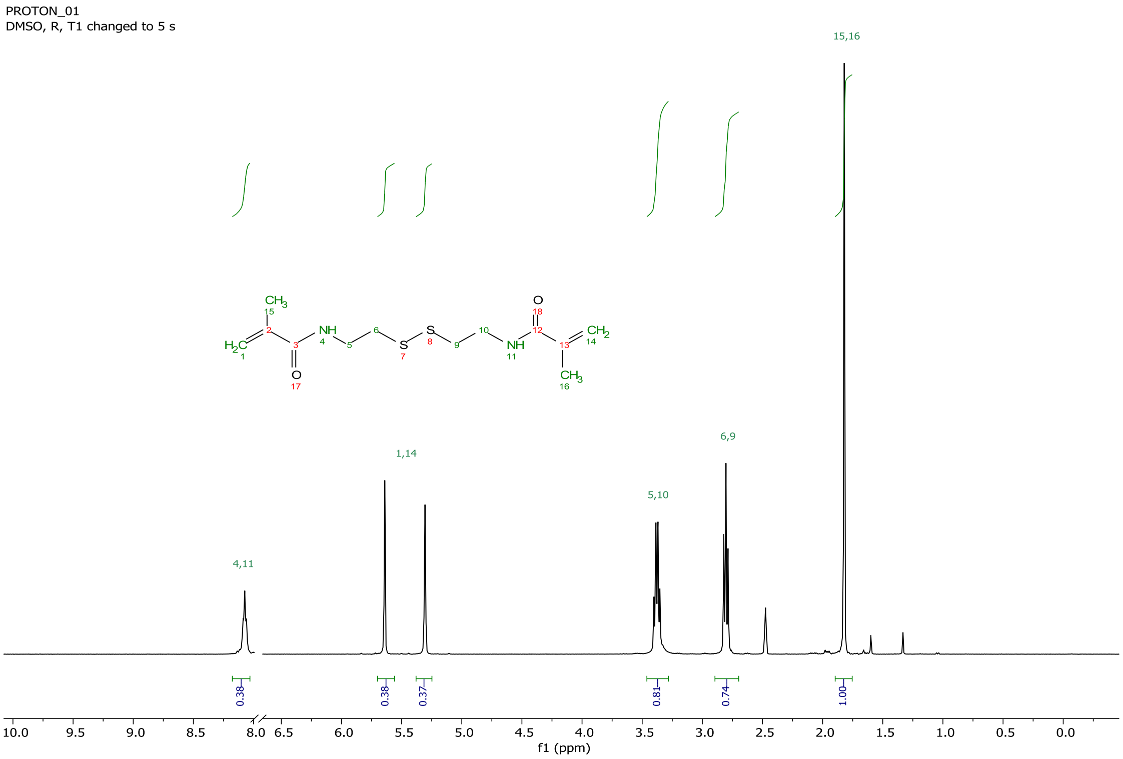

Figure S2. ^{1}H NMR spectra of the synthesized & purified BMAC.

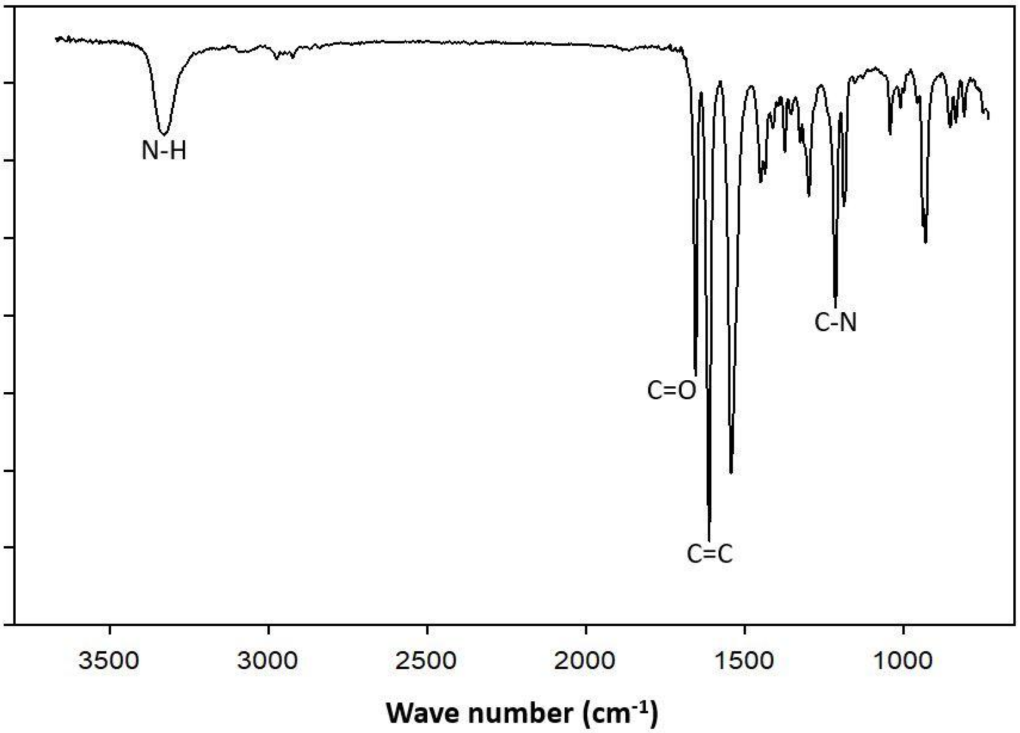

Figure S3. FTIR spectrum of synthesized and purified BMAC.

Monomers and the Initiator Purity Analysis by qNMR

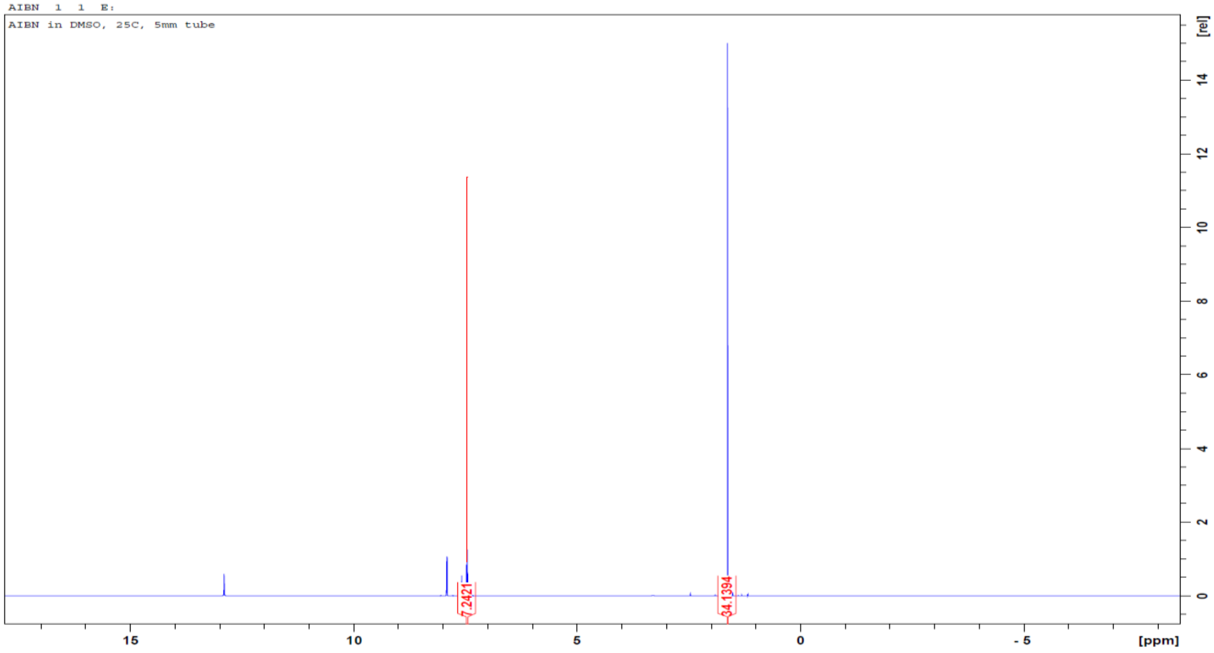

Figure S4. ^{1}H NMR spectrum of AIBN and benzoic acid standard.

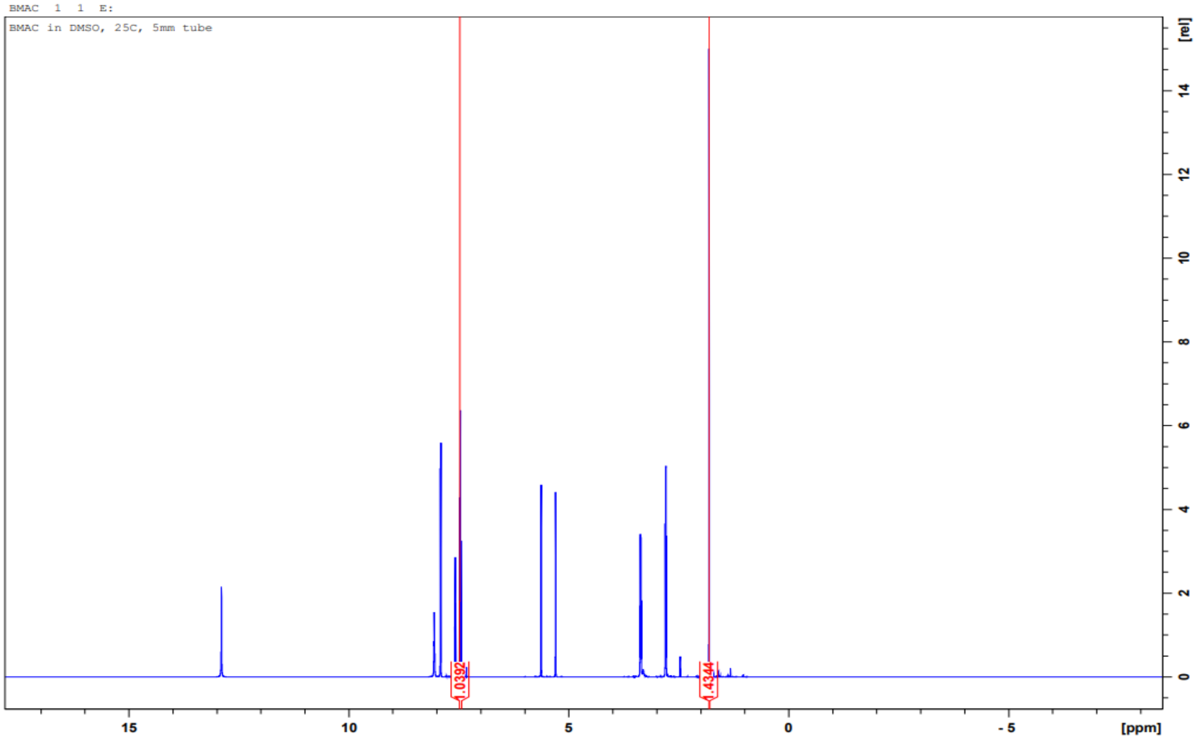

Figure S5. ^{1}H NMR spectrum of BMAC and benzoic acid standard.

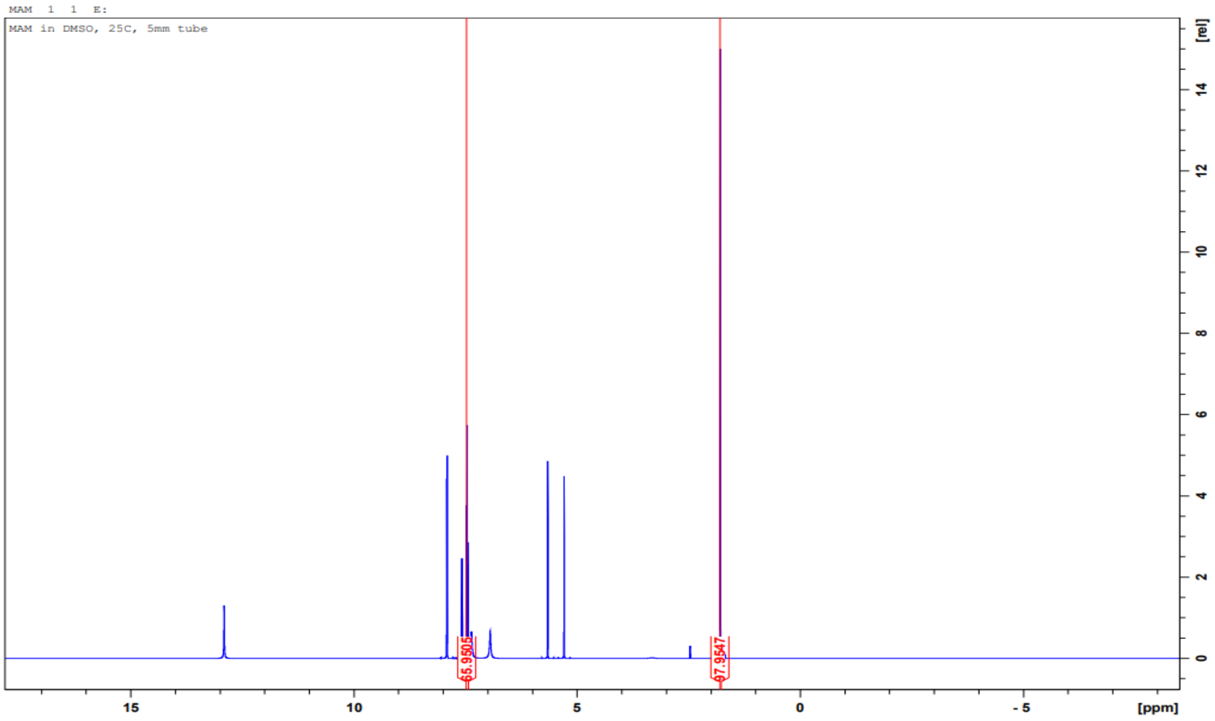

Figure S6. ^{1}H NMR spectrum of MAM and benzoic acid standard.

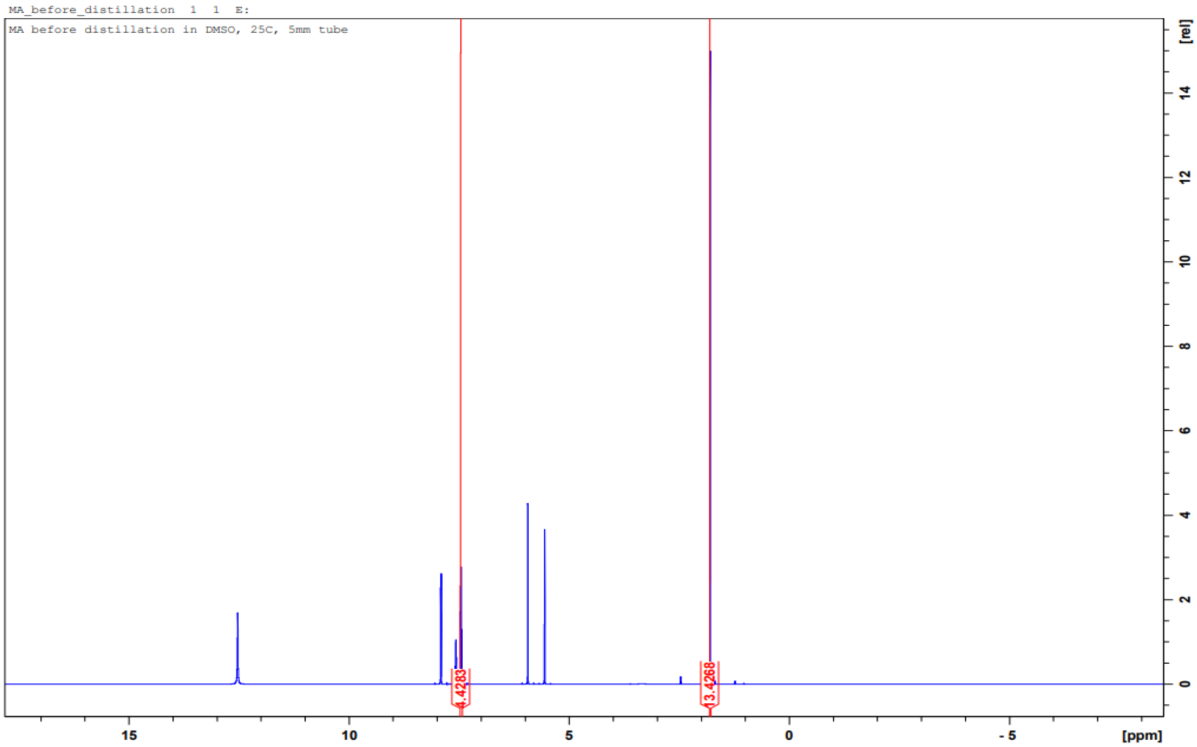

Figure S7. ^{1}H NMR spectrum of MAA, before distillation, and benzoic acid standard.

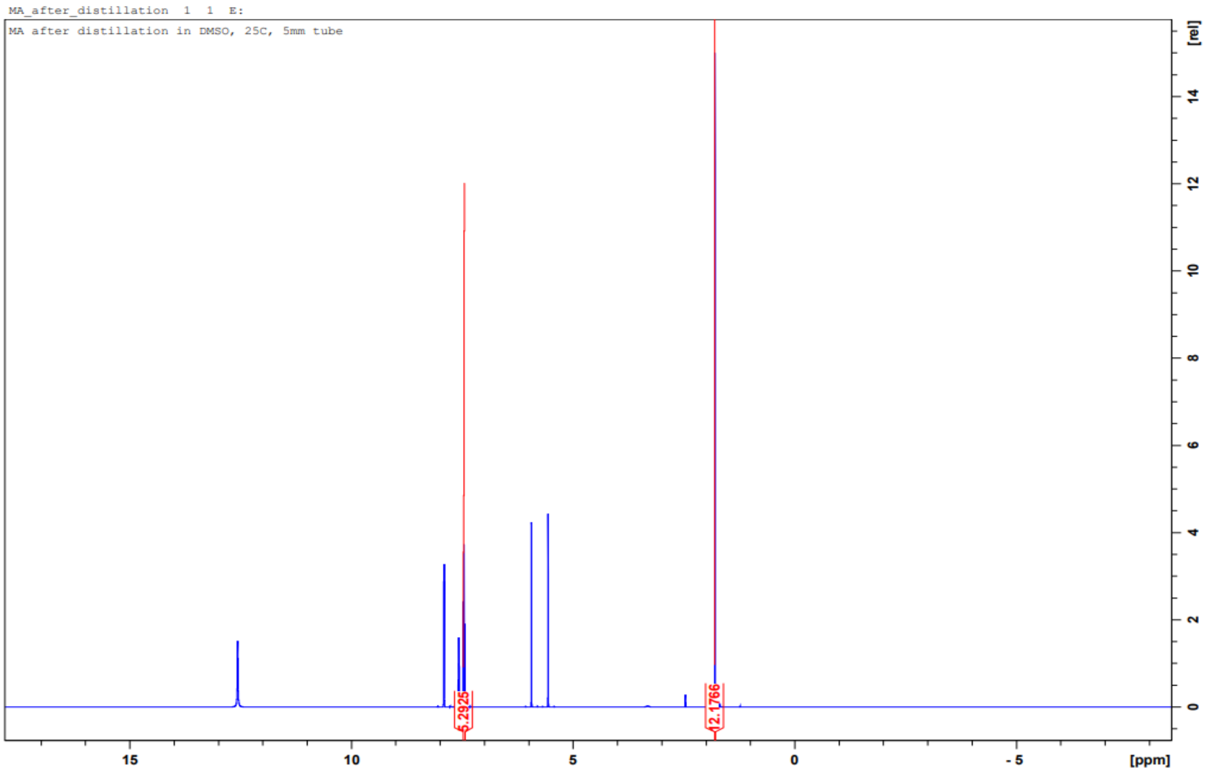

Figure S8. ^{1}H NMR spectrum of MAA, after distillation, and benzoic acid standard.

Calculations:

To calculate the purity of a sample, the following formula was used:

$$Px = \frac{Ix \times Nstd \times Mx \times Wstd}{Istd \times Nx \times Mstd \times Wx} \times 100$$

I: the integrated area

N: number of nuclei

M: molecular weight

W: gravimetric weight

Synthesis of 5 Batches of Poly(MAM-co-MAA-co-BMAC)

To a 20-ml glass vial, methacrylamide (3.07 g, 36 mmol), methacrylic acid (0.8 g, 9.3 mmol) and bis(methacryloyl)cystamine (0.13 g, 0.45 mmol) was added followed by addition of 8 ml of anhydrous DMF. The reactants were dissolved stock in the solvent, and nitrogen was purged in the solution for 30 min to remove dissolved oxygen molecules. A solution of AIBN initiator was made by dissolving 100 mg of AIBN in 5 ml DMF, and 0.25 ml of that solution was injected into

the reaction mixture. The solution was purged again with nitrogen for an additional 20 min at room temperature. The reaction mixture was incubated at 60 °C for 18 hours for copolymerization. At the end of the reaction time, the mixture was suspended in 200 ml of DI water, and the suspension was centrifuged at 2500 rpm for 10 min. The supernatant was discarded, and the remaining solid was suspended into DI water again. This procedure was repeated 4 more times. Next, the solid was suspended in DI water and the pH was adjusted to ~7.7 with 1M NaOH (10ml). To that solution was added a molar excess of DTT (0.69 g, 4.5 mmol) and the mixture was stirred for 18 hours at room temperature. At the end of the reaction, the mixture was observed to be less viscous. Next, 20 ml of HCl 1N was added, and the polymer precipitated. The precipitate was centrifuged at 4000 rpm for 6 min, followed by washing the remaining solid with nitrogen-purged 1mM HCl solution. The washing was repeated 3 more times, followed by washing with 1mM HCl/ETOH solution twice, and finally with 1 time nitrogen-bubbled HCl 1N. After centrifugation, the remaining copolymer was lyophilized overnight.

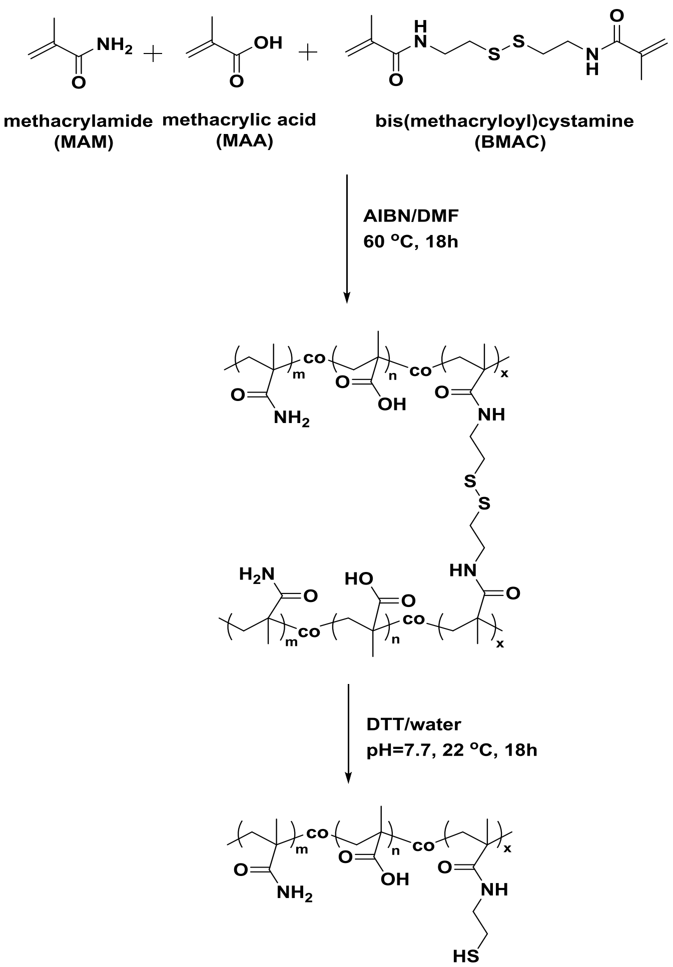

Figure S9. Reaction scheme of the copolymerization of MAM, MAA, and BMAC.

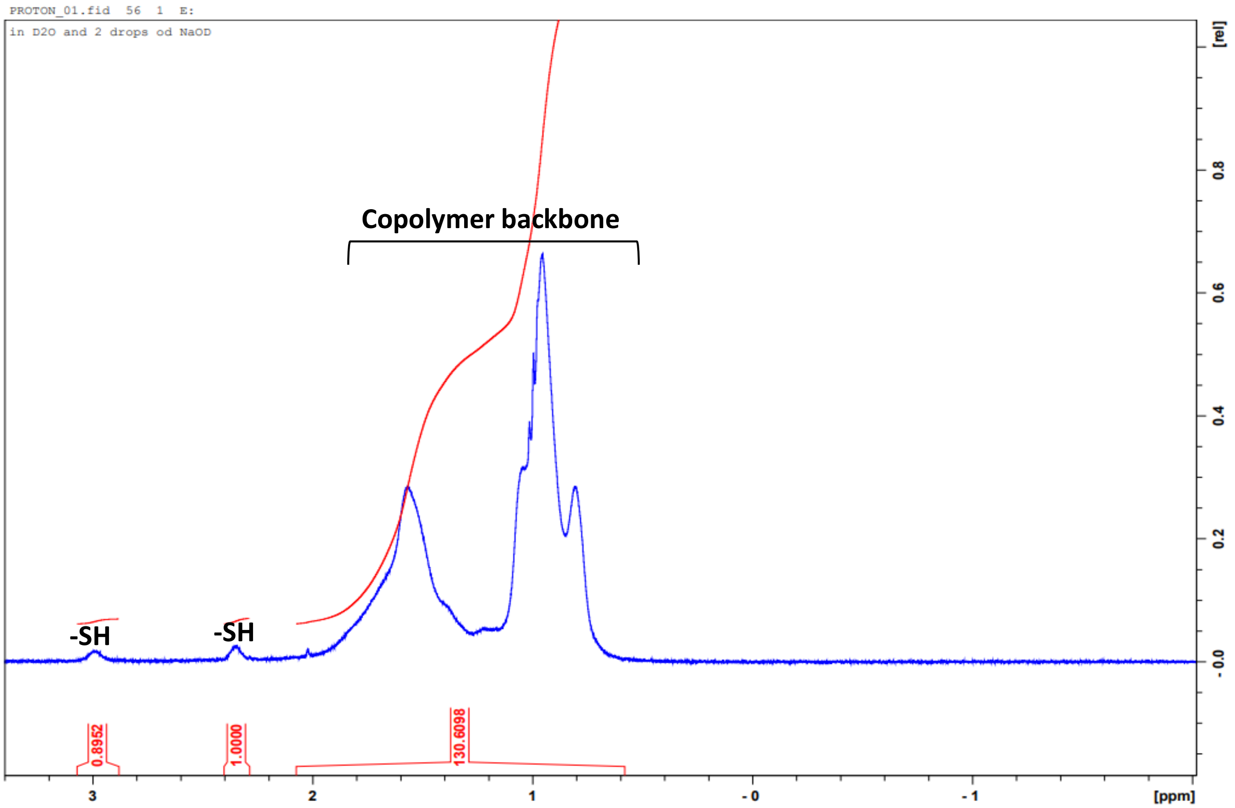

Figure S10. H[1] NMR of Poly(MAM-co-MAA-co-BMAC).

Synthesis of Thiolated Gellan

A solution of gellan 1.33 % w/v was made by stirring 4 g of gellan into 300 ml of Milli-Q water at 70° C. 30 min later, the pH was adjusted to 4.5, and the solution was cooled to 50° C. 1-Ethyl-3-(3-dimethylaminopropyl) carbodiimide (EDC; 0.76 g, 4.0 mmol), N-hydroxysuccinimide (NHS; 0.26 g, 2.2mmol) and cystamine dihydrochloride (0.7 g, 6.2mmol) was dissolved in 100 mL of Milli-Q water and added to the gellan solution while the temperature is maintained at 50°C. The mixture temperature was brought to RT within 30 min while under stirring. Gelation was observed at room temperature, and the stirring was allowed to continue for 4 more hours. Next, the pH of the reaction mixture was adjusted to 8-9 to terminate the reaction, and dialysis (MWCO = 12,000-14,000) against DI water was used to remove the unreacted reactants in addition to the reaction by-products. The dialysis was done for 3 days by changing the dialysate twice a day. The next step was to reduce the disulfide bond. This was done by first adjusting the pH of the reaction mixture to 7.5, followed by adding molar excess of DTT (4.8 g, 0.031 mol). The reaction was allowed to proceed for 3 hours, after which the thiolated gellan was purified by dialysis (MWCO 10K) against nitrogen-bubbled 1 mM Hydrochloric acid (8L x 9). Next, thiolated gellan was lyophilized and stored at -80 °C under nitrogen.

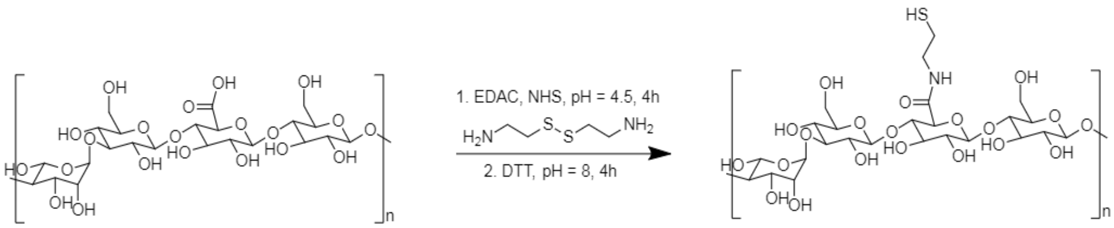

Figure S11. Reaction scheme of gellan amidation.

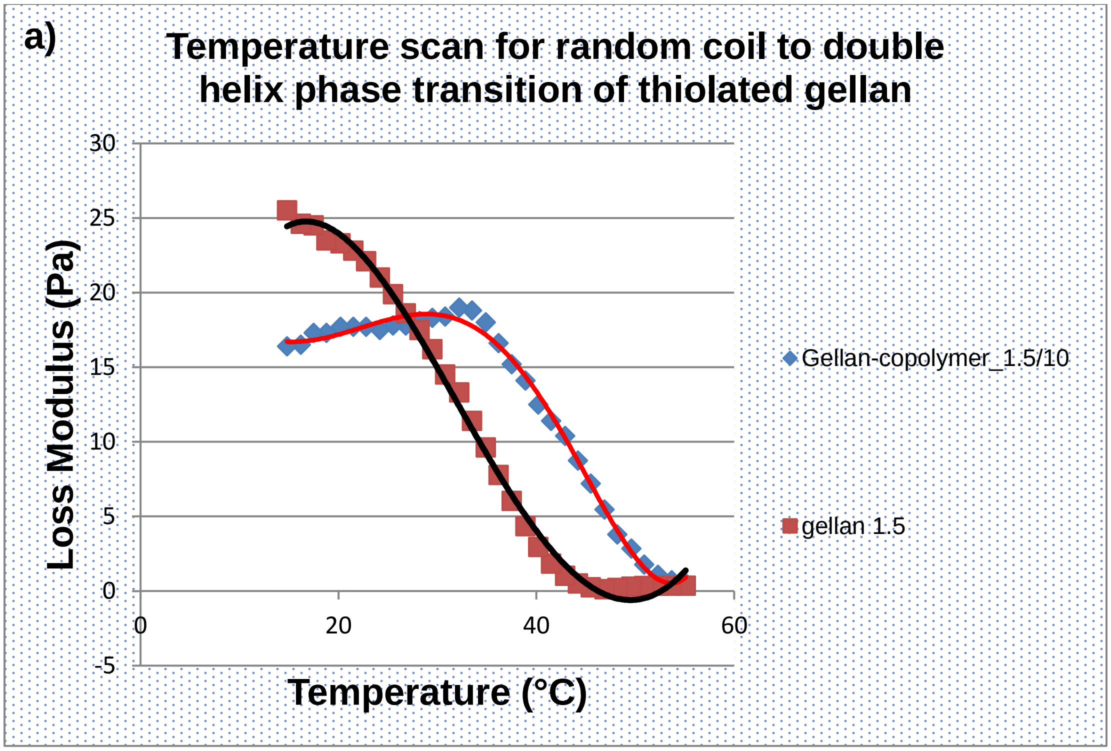

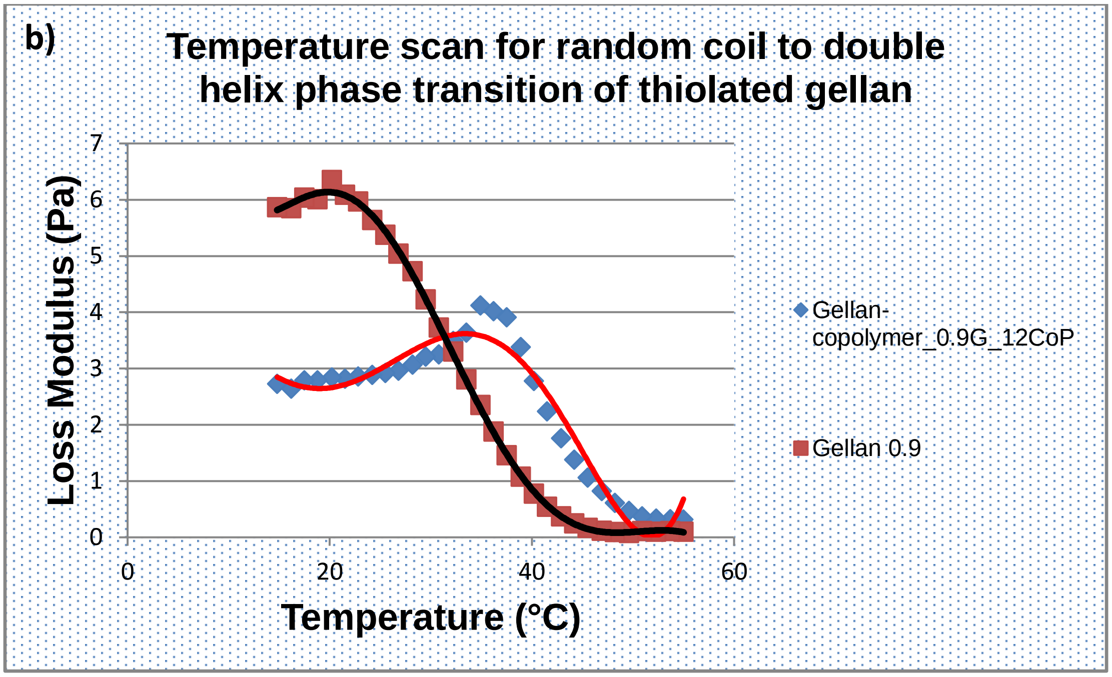

Figure S12-a) and b). Temperature scan of thiolated gellan and thiolated gellan-copolymer, formulation 1.5G_10CoP, and 0.9G_12CoP to determine the random coil to double helix phase transition of thiolated Gellan (taken as the middle point of the loss modulus increase). The midpoint of the curves in both mixtures is ~42°C.

Electroretinogram Analysis

In electroretinogram analysis, the a-wave is derived from the cones and rods of the outer photoreceptor layers. The a-wave shows the hyperpolarization of the photoreceptors due to the sodium ion channels closing in the outer membrane. While the b-wave is derived from the inner retina, it is mainly derived from the Muller and ON-bipolar cells. The b-wave is commonly used in the clinical and experimental analysis of human retinal function.
